# Gefitinib (IRESSA) sensitive lung cancer cell lines show phosphorylation of Akt without ligand stimulation

**DOI:** 10.1186/1471-2407-6-277

**Published:** 2006-12-06

**Authors:** Rintaro Noro, Akihiko Gemma, Seiji Kosaihira, Yutaka Kokubo, Mingwei Chen, Masahiro Seike, Kiyoko Kataoka, Kuniko Matsuda, Tetsuya Okano, Yuji Minegishi, Akinobu Yoshimura, Shoji Kudoh

**Affiliations:** 1 Department of Pulmonary Medicine/Infection and Oncology, Nippon Medical School, 1-1-5 Sendagi, Bunkyo-ku, Tokyo 113-8602, Japan

## Abstract

**Background:**

Phase III trials evaluating the efficacy of gefitinib (IRESSA) in non-small cell lung cancer (NSCLC) lend support to the need for improved patient selection in terms of gefitinib use. Mutation of the epidermal growth factor receptor (*EGFR*) gene is reported to be associated with clinical responsiveness to gefitinib. However, gefitinib-sensitive and prolonged stable-disease-defined tumors without *EGFR *gene mutation have also been reported.

**Methods:**

To identify other key factors involved in gefitinib sensitivity, we analyzed the protein expression of molecules within the EGFR family, PI3K-Akt and Ras/MEK/Erk pathways and examined the sensitivity to gefitinib using the MTT cell proliferation assay in 23 lung cancer cell lines.

**Results:**

We identified one highly sensitive cell line (PC9), eight cell lines displaying intermediate-sensitivity, and 14 resistant cell lines. Only PC9 and PC14 (intermediate-sensitivity) displayed an *EGFR *gene mutation including amplification. Eight out of the nine cell lines showing sensitivity had Akt phosphorylation without ligand stimulation, while only three out of the 14 resistant lines displayed this characteristic (*P *= 0.0059). Furthermore, the ratio of phosphor-Akt/total Akt in sensitive cells was higher than that observed in resistant cells (*P *= 0.0016). Akt phosphorylation was partially inhibited by gefitinib in all sensitive cell lines.

**Conclusion:**

These results suggest that Akt phosphorylation without ligand stimulation may play a key signaling role in gefitinib sensitivity, especially intermediate-sensitivity. In addition, expression analyses of the EGFR family, *EGFR *gene mutation, and FISH (fluorescence *in situ *hybridization) analyses showed that the phosphorylated state of EGFR and Akt might be a useful clinical marker of Akt activation without ligand stimulation, in addition to *EGFR *gene mutation and amplification, particularly in adenocarcinomas.

## Background

Gefitinib(IRESSA) [4-(3-chloro-4-fluoroanilino)-7-methoxy-6-(3-morpholinopropoxy)-quinazoline] is an orally active, epidermal growth factor receptor (EGFR) tyrosine kinase inhibitor that inhibits EGFR signaling [1,2]. IDEAL (IRESSA Dose Evaluation in Advanced Lung cancer) 1 and 2 were randomized Phase II trials in patients with non-small cell lung cancer (NSCLC) refractory to platinum-based chemotherapy. These trials demonstrated that gefitinib was active and generally well tolerated [3,4]. However, ISEL (IRESSA Survival Evaluation in Lung cancer), a Phase III trial evaluating the efficacy of gefitinib compared to best supportive care for refractory or recurrent NSCLC, failed to demonstrate a significant survival benefit, except in an Eastern subpopulation and in those who had never smoked [5]. These data indicate that improved patient selection and combination strategies are probably required to maximize the benefits of using this targeted therapy.

Gefitinib exerts its antitumor activity through the inhibition of EGFR tyrosine kinase; however, this activity does not significantly correlate with the level of EGFR expression by the tumor cell [6]. Recent reports have shown that there are differences between gefitinib responders and non-responders in the frequency of activating mutations in the *EGFR *gene, which suggests that such mutations might be predictive markers for sensitivity to gefitinib [7,8]. *EGFR *gene amplification, as detected by fluorescence in situ hybridization (FISH), was also reported to be a predictive marker [9]. However, there are known to be gefitinib-sensitive and intermediate-sensitive tumors that have no activating mutations in the *EGFR *gene, including gene amplification. Thus, it is likely that other factors in lung cancer cells may sensitize cells to gefitinib in addition to *EGFR *gene mutation and the amplification detected by FISH. In order to identify additional key molecules involved in gefitinib resistance, we examined the sensitivity to gefitinib of 23 lung cancer cell lines using the 3- [4,5-dimethylthiazol-2-yl]-2,5-diphenyltetrazolium bromide (MTT) cell proliferation assay and identified sensitive, intermediate-sensitive, and resistant lung cancer cell lines. A few reports have suggested that small cell lung cancer(SCLC) is responsive to gefitinib [10,11]. Tanno *et al *reported that MAPK, a downstream effector of the EGFR was inhibited by gefitinib in SCLC cell lines that expressed the EGFR even at a very low level[11]. SCLC cell lines were included in our series. We also analyzed the genomic status of the *EGFR *gene mutation and *EGFR *gene amplification, as well as the protein expression level of key molecules in the EGFR family (EGFR, Her2, Her3), PI3K-Akt (Akt and phosphatase and tensin homolog [PTEN]) and Ras/MEK/Erk pathways (p44/42 mitogen-activated protein [MAP] kinase, p38 MAP kinase) which act downstream of EGFR. We correlated the cytotoxic activity of gefitinib in our 23 lung tumor cell lines to the corresponding expression patterns of these proteins.

## Methods

### Cell Lines

Twenty-three lung cancer cell lines were used in this study. They comprised: 10 adenocarcinoma cell lines (ABC-1, A549, PC3, PC7, RERF-LCMS, RERF-LCKJ, LCD, LCOK, PC9, and PC14), eight squamous-cell carcinoma cell lines (QG56, EBC-1, LK-2, LC-1/sq, PC1, RERF-LCAI, PC10, and SQ5), and five small-cell carcinoma cell lines (NCI-H69, SBC3, NCI-N231, Lu135, and MS-1). The Lu135 cell line was provided by Y. Shimosato and T. Terasaki, and the LCD and LCOK cell lines by S. Hirohashi (National Cancer Center Research Institute, Tokyo, Japan). The NCI-N231, A549, and NCI-H69 cell lines were obtained from the American Type Culture Collection (Rockville, MD) [12,13]. The PC1, PC3, PC7, PC9, PC10, PC14, and QG56 cell lines were obtained from IBL (Gunma, Japan). The RERF-LCKJ, SQ5, LC-1/sq, RERF-LCAI, and MS-1 cell lines were obtained from RIKEN Cell Bank (Ibaragi, Japan). The ABC-1, RERF-LCMS, LK-2, EBC-1, and SBC3 cell lines were obtained from the Health Science Research Resources Bank (Osaka, Japan).

In order to determine the activation of the members downstream of EGFR without ligand stimulation, all cell lines were separately cultured in serum-containing (+) and serum-free (-) conditions for 24 h.

### Drugs and Growth-Inhibition Assay

Gefitinib was provided by AstraZeneca and dissolved in dimethyl sulfoxide (DMSO) for *in vitro *studies. We used the colorimetric MTT assay to examine the activity of gefitinib against all 23 lung cancer cell lines as previously reported [14]. Cell suspensions (200 *μ*l, 10^5 ^cells/ml) were seeded into 96-well microtiter plates and 10 *μ*l of drug solution added, at various concentrations. After incubation for 72 h at 37°C, 20 *μ*l of MTT solution (5 mg/ml in phosphate buffered saline [PBS]) was added to each well and incubation then continued for a further 4 h at 37°C. The IC_50 _value was defined as the concentration of gefitinib needed for a 50% reduction in absorbance (560 nm) based on cell growth curves.

### Western Blot Analysis

Western blot analysis was performed as previously described [15]. The membranes were first incubated overnight at 4°C with antibody specific for the following primary antibodies: PTEN (Santa Cruz Biotechnology, Santa Cruz, CA) and Akt, phospho-Akt (Ser473), p42/44 MAP kinase and phospho-p44/42 MAP kinase (Thr202/Tyr204), p38 MAP kinase and phospho-p38 MAP kinase (Thr180/Tyr182), β-actin, EGFR, and phospho-EGFR (Y1068), Her2 and Her3 [all from Cell Signaling Technology, Beverly, MA]. The membranes were then incubated with peroxidase-conjugated secondary antibodies and protein was detected with enhanced chemiluminescence (ECL) Western blotting detection reagents (Amersham, Buckinghamshire, UK) [16,17]. These images were quantified by measuring signal intensity using National Institute of Health (NIH) Image (ImageJ1.32j). The ratios of phospho-EGFR/EGFR, Her2/β-actin, Her3/β-actin, phospho-Akt/Akt, PTEN/β-actin, phospho-p44/42 MAP kinase/p44/42 MAP kinase, and phospho-p38 MAP kinase/p38 MAP kinase were calculated from cells grown in both serum-containing (+) and serum-free (-) conditions.

### Polymerase Chain Reaction Single Strand Conformation Polymorphism Analyses and Sequencing of Genomic DNA

Genomic DNA samples were obtained from each cell line by proteinase K treatment and phenol chloroform extraction using standard protocols [17]. From each genomic DNA sample, exons 18, 19 and 21 of the *EGFR *gene and exon 1 and 2 of the *K-Ras *gene were amplified separately with the relevant polymerase chain reaction (PCR) primers [7,18] using the Gene Amp XL PCR kit (Perkin Elmer/Roche, Branchburg, NJ). PCR conditions for genomic DNA analysis were as follows: 40 cycles at 94°C for 40 seconds, 60°C for 30 seconds, and 68°C for 90 seconds, followed by 68°C for 8 minutes. Fluorescein-isothiocyanate (FITC)-labeled PCR products were denatured, cooled on ice, and loaded on neutral 6% polyacrylamide gels with or without 5% (vol/vol) glycerol, as described previously [13]. After electrophoresis, the gels were analyzed with the FluorImager 595 (Molecular Dynamics Inc., Sunnyvale, CA). Each DNA sample was examined with three primer pair combinations, with the M13 sequence (TGTAAAACGACGACGGCCAGT) added in each case to the appropriate primer. PCR analysis was performed as described above, with the resulting products being purified and sequenced by fluorescence-based automated sequencing (Perkin Elmer/Applied Biosystems, Foster City, CA).

### FISH Analysis

Cells were fixed on slides in Carnoy's fluid (60% ethanol; 30% chloroform; 10% acetic acid) and incubated for 30 min at 60°C and 30 min at 37°C in 2 × sodium chloride sodium citrate solution (SSC)/0.1%Tween20 followed by ethanol dehydration. The LSI EGFR/CEP7 SpectrumGreen Probe (Vysis Downers Grove, IL) was applied to the slides [9] which were then incubated for 5 min at 75°C for the codenaturation of probe and chromosomal DNA. Hybridization proceeded overnight at 37°C, after which the slides were washed in 50% formamide/2 × SSC for 15 min, 2 × SSC for 10 min and 2 × SSC/0.1%Tween29 for 5 min at 47°C. The chromatin was counterstained with 4'6'-diamidino-2-phenylindole (DAPI) (Roche). The frequency of tumor cells with specific copy numbers of the *EGFR *gene (red signal) and chromosome 7 (green signal) were estimated under a BX61 Olympus fluorescence microscope in a minimum of 200 nuclei. A gene amplification was defined as gene/chromosome per cell ratio >2 or >15 copies of EGFR per cell in >10% of analyzed cells. *EGFR *gene copy numbers were counted and were classified into six FISH strata by Hirsh's criteria [9,19].

### Exposure to Gefitinib

Lung cancer cell lines were serum-starved for 24 h and treated with various concentrations of gefitinib (0, 0.1, 1 and 10 *μ*M/ml) for 2 h before exposure to 10 ng/ml epidermal growth factor (EGF; [BA-53, Santa Cruz Biotechnology]) for 5 min in order to evaluate the effect of gefitinib on the PI3K-Akt and Ras/MEK/Erk pathways. These images were quantified by measuring signal intensity using NIH Image (ImageJ1.32j).

### Statistical Analysis

The Chi-square test was used to evaluate the relationship between gefitinib sensitivity and activation of the molecules in the EGFR family, PI3K-Akt and Ras/MEK/Erk pathways. The cut-off value of signal intensity measured by NIH Image was defined by Receiever Operating Characteristics (ROC)curve. The expression ratio of phospho-EGFR/EGFR, Her2/*β*-actin, Her3/*β*-actin, phospho-Akt/Akt, phospho-p44/42 MAP kinase/p44/42 MAP kinase, phospho-p38 MAP kinase/p38 MAP kinase and PTEN/*β*-actin was compared between gefitinib-resistant and gefitinib-sensitive cell lines using the Mann-Whitney test. In addition, the correlations between PTEN expression and phosphorylation of Akt were examined by Spearman's rank order coefficient across all 23 cell lines. *P *< 0.05 was considered to be statistically significant.

## Results

### Effect of Gefitinib on Cell Growth *In Vitro*

**Figure 1:**
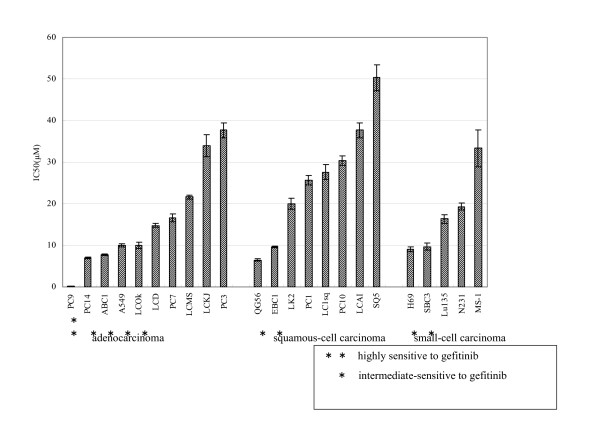
IC_50 _values for 23 lung cancer cell lines using the MTT assay. Cell lines were classified as: sensitive (IC_50_≤1 *μ*M), intermediate-sensitive (1 *μ*M < IC_50_≤10 *μ*M), and resistant (10 *μ*M <IC_50_) to gefitinib. The bars represent the IC_50 _and standard deviation (SD) obtained in three indpendent MTT assays. *Point*: mean of three independent experiments, bar: ± SD.

The IC_50 _values of gefitinib for the 23 lung cancer cell lines, as determined by MTT assay, are summarized in Fig. 1. Only the PC9 cell line had an IC_50 _of <1 *μ*mol/L (highly-sensitive), 14 cell lineshad an IC_50 _of >10 *μ*mol/L (resistant), and the remaining eight had an IC_50 _of 1 to 10 *μ*mol/L (intermediate-sensitive).

### Expression and Phosphorylation Status of EGFR, Her2, Her3, Akt, p44/42, p38 MAP kinase, and PTEN in Gefitinib-sensitive Versus Gefitinib-resistant Cell Lines

**Figure 2:**
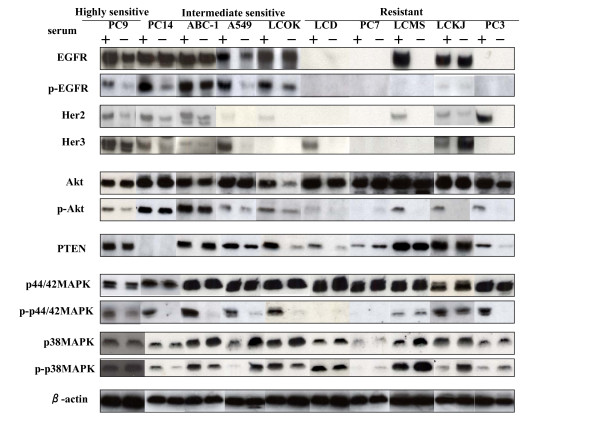
Western blot analyses: protein expression and phosphorylation of epidermal growth factor receptor (EGFR), Akt, PTEN, Her2, Her3, p44/42 mitogen activated protein kinase (MAPK), and p38 MAPK in adenocarcinoma cell lines cultured for 24 h with (+) or without (-) serum.

**Figure 3:**
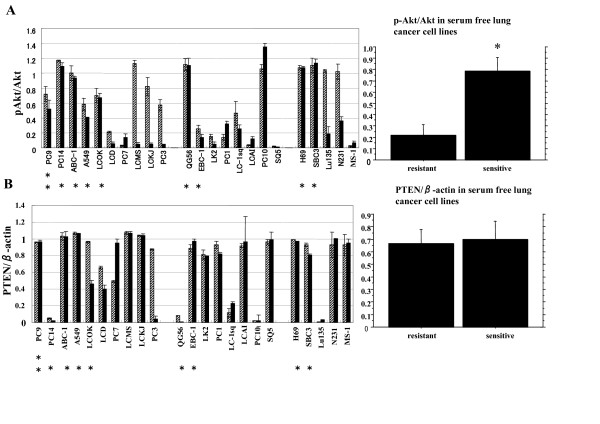
Summary of expression of PTEN and phosphorylation of Akt in 23 lung cancer cell lines grown under serum-containing (cross bars) and serum-free (black bars) conditions. (A) The ratio of phosphorylated Akt signal/total Akt signal (pAkt/Akt); (B) the ratio of PTEN signal/β-actin signal; The density of expression of each of the molecules was measured by NIH Image. *Point*: mean of three independent experiment; bars, ± standard deviation (SD). The expression ratio of the molecules in serum-free conditions was compared between gefitinib-resistant and gefitinib-sensitive cell lines using the Mann-Whitney test; *point*, mean, bars, ± standard error (SE). *P < 0.05 was considered to be statistically significant.

**Figure 4:**
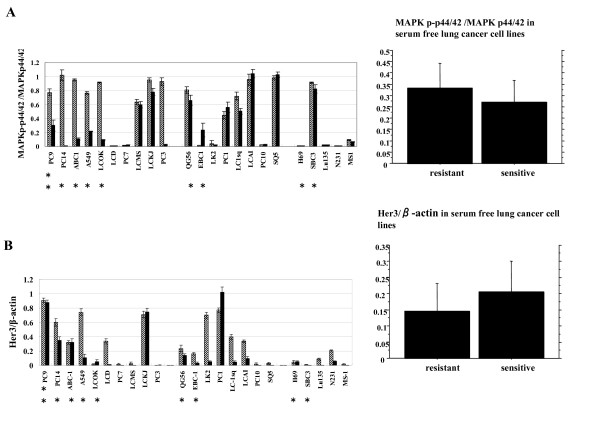
Summary of expression of Her3 and phosphorylation of p44/42 MAPK in 23 lung cancer cell lines grown under serum-containing (cross bars) and serum-free (black bars) conditions. (A) the ratio of phosphorylated p44/42 MAPK signal/total p44/42MAPK signal (MAPKp-p44/42/MAPKp44/42); (B) the ratio of Her3 signal/β-actin signal; The density of expression of each of the molecules was measured by NIH Image. *Point*: mean of three independent experiment; bars, ± standard deviation (SD). The expression ratio of the molecules in serum-free conditions was compared between gefitinib-resistant and gefitinib-sensitive cell lines using the Mann-Whitney test; *point*, mean, bars, ± standard error (SE). *P < 0.05 was considered to be statistically significant.

**Figure 5:**
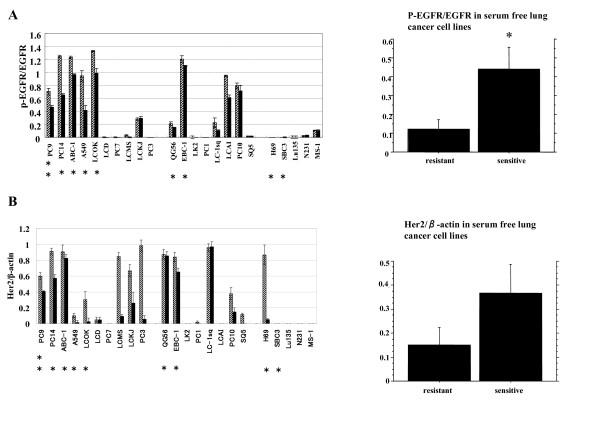
Summary of expression of Her2 and phosphorylation of EGFR in 23 lung cancer cell lines grown under serum-containing (cross bars) and serum-free (black bars) conditions. (A) the ratio of phosphorylated EGFR signal/EGFR signal; (B) the ratio of Her2 signal/β-actin signal. The density of expression of each of the molecules was measured by NIH Image. *Point*: mean of three independent experiment; bars, ± standard deviation (SD). The expression ratio of the molecules in serum-free conditions was compared between gefitinib-resistant and gefitinib-sensitive cell lines using the Mann-Whitney test; *point*, mean, bars, ± standard error (SE). *P < 0.05 was considered to be statistically significant.

The protein expression levels and phosphorylation status of EGFR, Her2, Her3, Akt, p44/42, p38 MAP kinase, and PTEN were analyzed in all 23 lung cancer cell lines by measuring the signal intensity using NIH Image (ImageJ1.32j) (Figs. 2, 3, 4 and 5). To evaluate activation without ligand stimulation, the cell lines were cultured in serum-containing (+) and serum-free (-) conditions. The ratios of phospho-EGFR/EGFR, Her2/*β*-actin, Her3/*β*-actin, phospho-Akt/Akt, PTEN/β-actin, phospho-p44/42 MAP kinase/p44/42 MAP kinase, and phospho-p38 MAP kinase/p38 MAP kinase were calculated in cells grown under both conditions. Across the entire cohort of 23 lung cancer cell lines, those showing sensitivity to gefitinib exhibited greater phosphorylation of Akt and EGFR without ligand stimulation than gefitinib-resistant cell lines, according to the Mann-Whitney test (*P *= 0.0016, *P *= 0.0274, respectively) (Figs. 3 and 5). Furthermore, the ratio of phospho-Akt/total Akt in intermediate-sensitive cells was higher than that observed in resistant cells (*P *= 0.003). There was no statistical difference in the phosphorylation of p44/42 MAP kinase, p38 MAP kinase without ligand stimulation, and expression of PTEN, Her2 and Her3 (Mann-Whitney test: PTEN: *P *= 0.8; p-p44/42: *P *= 0.8; p-p38: *P *= 0.45; Her2:*P *= 0.052; Her3:*P *= 0.08, respectively) (Fig. 3, 4 and 5). As shown in Table 1, gefitinib-sensitive cells frequently expressed phospho-Akt, phospho-EGFR and Her2 with ligand stimulation (P = 0.0059, *P *= 0.0319; *P *= 0.0344, Chi-square test). In particular, cell lines characterized by phosphorylation of Akt without ligand stimulation had a greater than 29 times probability of being sensitive to gefitinib than other cells. Interestingly, sensitive cells, especially in the adenocarcinoma cell lines, had more Akt phosphorylation with EGFR phosphorylation than resistant cells in serum-containing conditions (Chi-square test: p = 0.034: total, p = 0.0114: in adenocarcinoma) (Table 1). In eight squamous cell carcinoma cell lines, PTEN expression seemed to be inversely correlated with phosphorylation of Akt (Spearman's rank order coefficient: r = -0.76, P = 0.04) (Fig. 3). However, in the other histological types of cells, PTEN expression did not correlate with phosphorylation of Akt (Spearman's rank order coefficient: adenocarcinomas: *r *= -0.08, *P *= 0.17, small-cell carcinomas: *r *= 0.1, *P *= 0.84).

### Mutation Analyses of the EGFR *Gene *and *K-Ras *Gene

Correlations have been reported between *EGFR *mutation and clinical responsiveness to gefitinib [7,8]. Therefore, exons 18, 19, and 21 in the *EGFR *gene were sequenced from 23 lung cancer cell lines, in order to assess mutations in the tyrosine kinase domain. The PC9 cell line, which was sensitive to gefitinib, had an in-frame deletion within the EGFR gene, removing amino acids 746 through 750 (delE746-A750) within the activation loop of the tyrosine kinase that flanks the ATP cleft. The other lung cancer cell lines did not display any *EGFR *gene mutations in these regions. A549(intermediate-sensitive) and LCKJ(resistant) cell lines had a point mutation(*G12S*) in codon 12 evaluated by *K-ras *gene mutational analyses.

### FISH Analysis

**Figure 6:**
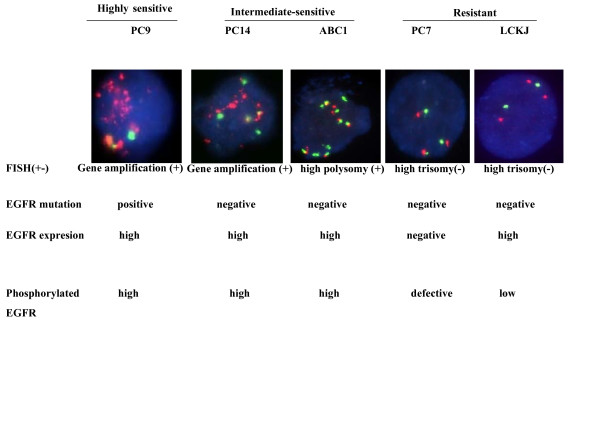
Fluorescence *in situ *hybridization (FISH) analyses of lung cancer cell lines, and EGFR mutations and expression. EGFR gene copy numbers were counted and were classified into six FISH strata [9,19]. EGFR gene amplification was detected in PC9 and PC14 cells. In the ABC1 cell line, the EGFR gene copy number was five in over 40% of cells (high polysomy:FISH negative); in the PC7 and LCKJ cell lines, the EGFR gene copy number was three in over 40% of cells (high trisomy:FISH negative).

Genomic gains of the *EGFR *gene were examined by FISH analysis. PC9 (highly-sensitive cell) with *EGFR *mutation, and PC14 (intermediate-sensitive cell) both had *EGFR *gene amplification (Fig. 6) [9,19]. There were high levels of expression and phosphorylation of EGFR in PC9 and PC14.

### Effect of Gefitinib on the Phosphorylation of EGFR, Akt, p44/42 MAP kinase, and p38 MAP kinase

**Figure 7:**
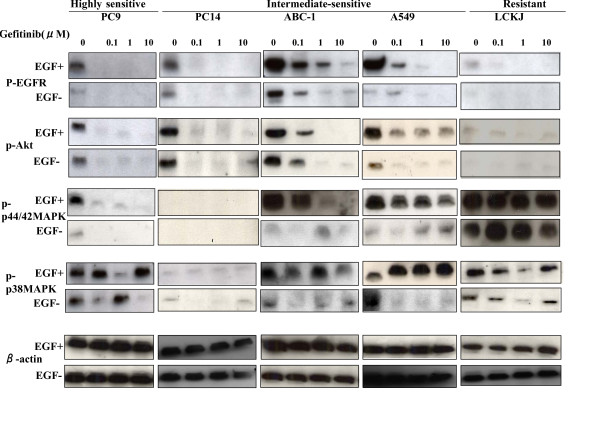
Lung cancer cell lines: Effect of gefitinib on phosphorylated EGFR(p-EGFR), phosphorylated Akt (p-Akt) and the molecules in the Ras/MEK/Erk pathway with or without EGF.

**Figure 8:**
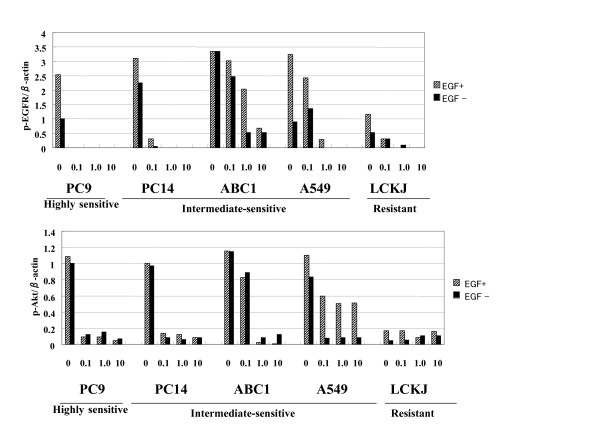
Summary of the effect of gefitinib on the molecules downstream of EGFR (Fig.7.). These images were quantified by measuring signal intensity using NIH Image (ImageJ1.32j).

The effect of gefitinib was investigated on the phosphorylation of EGFR, Akt, p44/42 MAP kinase, and p38 MAP kinase, in serum-starved, gefitinib-pretreated lung cancer cells (Figs. 7 and 8). Akt and EGFR were phosphorylated without ligand stimulation in the highly gefitinib-sensitive cell line PC9, which has an EGFR gene mutation, as well as intermediate-sensitive cell line. Akt and EGFR phosphorylation were inhibited by gefitinib in sensitive cell lines (Figs. 7 and 8). With EGF stimulation, phosphorylation of Akt increased further in A549 and PC9 cells, but not in PC14 and ABC-1 cells. In the resistant cell lines, Akt was phosphorylated through gefitinib treatment, although EGFR phosphorylation was inhibited (Figs. 7 and 8). In PC9 cells, phosphorylation of p44/42 MAP kinase was inhibited at low concentrations of gefitinib. However, in cells showing intermediate sensitivity to gefitinib, phosphorylation of p44/42 MAP kinase was not clearly inhibited, either in the presence or absence of EGF. Phosphorylation of p38 MAP kinase was not clearly inhibited in anyof the lung cancer cell lines.

Eight of the nine lines showing sensitivity to gefitinib had Akt phosphorylation without ligand stimulation, while only three of the 14 resistant lines displayed this characteristic. Furthermore, the ratio of phospho-Akt to total Akt in sensitive cells was higher than that observed in resistant cells. Akt phosphorylation was partially inhibited by gefitinib in highly and intermediate-sensitive cell lines. These results suggest that Akt activation without ligand stimulation may play a key signaling role in gefitinib sensitivity.

## Discussion

Clinically, gefitinib-sensitive tumors have been observed that contain no evidence of activating mutations in the *EGFR *gene [20]. We examined the sensitivity of 23 lung cancer cell lines to gefitinib using the MTT cell proliferation assay and identified one highly gefitinib-sensitive lung cancer cell line (PC9), eight intermediate-sensitive lung cancer cell lines, and 14 resistant lung cancer cell lines. The IC_50 _value of the PC9 cell line was about one-sixth of the clinical dose. Only the PC9 cell line displayed a mutational event in the *EGFR *gene: a 15 bp deletion in exon 19. The IC_50 _values in the intermediate-sensitive cell lines ranged between 1 and 10 *μ*M, which was similar to a previous report [21]. In our study, the IC_50 _value in A549 cells was 10 *μ*M, which has been previously reported in a xenograft model as sensitive to gefitinib [22,23]. These values are higher than the maximum serum concentration of gefitinib observed in patients (~1 *μ*M). Nevertheless, *in vitro *studies occasionally do not correlate with *in vivo *work. However, these differences in this study mainly seem to depend on the exposure time to gefitinib. In the xenograft study, A549 was sensitive in gefitinib-intake for 35 days. In our study, Akt phosphorylation was partially inhibited by gefitinib (<1 *μ*M) in intermediate-sensitive cell lines, whereas their IC_50 _value ranged between 1 and 10 *μ*m.

In this study, cancer cell lines showing sensitivity to gefitinib exhibited more phosphorylation of Akt and EGFR without ligand stimulationthan gefitinib-resistant cell lines (Mann-Whitney test:*P *= 0.0016, *P *= 0.0274, respectively). Sensitive cells frequently had phospho-Akt and phospho-EGFR without ligand stimulation (*P *= 0.0059, *P *= 0.0319; Chi-square test). This is the first report suggesting that unstimulated phosphorylation of Akt seems to have a strong correlation with gefitinib sensitivity, especially, intermediate sensitivity. Unstimulated phosphorylation of Akt seems to be mainly due to constitutive activation of the Akt signaling pathway. Cappuzzo *et al *have reported that patients with phospho-Akt-positive tumors who received gefitinib had a better response rate in terms of stable disease, disease control rate, and time to disease progression than patients with phospho-Akt-negative tumors [24]. Our results support these clinical findings. In our study, Akt phosphorylation was inhibited by gefitinib in all these cell lines. In this situation, there remains a question whether Akt is really so central in determining the sensitivity to gefitinib or if it is just a downstream molecule that is sensing activation of other upstream molecules. Amann *et al *reported that the NSCLC cell line H1819, which does not have an *EGFR *mutation, but shows high expression levels of EGFR, ErbB2, and ErbB3, showed intermediate sensitivity to tyrosine kinase inhibitors [25]. They also reported that, in this cell line, Akt was constitutively phosphorylated, but remained prone to inhibition by an EGFR-directed tyrosine kinase inhibitor. They suggested that, in addition to *EGFR *gene mutation, other factors, such as high expression levels of ErbB family members, might constitutively activate Akt and sensitize cells to EGFR inhibitors [25]. The EGFR family (EGFR, Her2 and Her3) operates a complex the signal transduction of its downstream with makes the formation of each receptor homo- or heterodimerzation and transducecascade through PI3K-Akt and Ras-Erk-MAPK pathways. Over expression of Her2 has been shown to promote the constitutive phosphorylation of EGFR and to delay and prolong the phosphorylation of EGFR [26]. In our study, there were statistically significant differences in Her2 expression between gefitinib-sensitive and resistant cells (P = 0.0344; Chi-square test). Unstimulated phosphorylation of Akt might be a hallmark of sensing activation of other upstream molecules.

In highly- and intermediate-sensitive cells, Akt was phosphorylated without ligand stimulation. Clinical markers of Akt activation without ligand stimulation should be looked for. The eight cell lines with Akt phosphorylation without ligand stimulation consisted of: five adenocarcinomas, one squamous cell carcinoma, and two small-cell carcinomas. All five adenocarcinomas had EGFR phosphorylation (Figs. 2, 3 and 5). In all the adenocarcinoma lines, the phosphorylation state of EGFR was predictive of Akt phosphorylation without ligands stimulation (Figs. 2, 3 and 5). Both PC9 (gefitinib-sensitive cell line) with EGFR mutation, and PC14 (intermediate-sensitive cell line) had EGFR gene amplification. These lines had the EGFR and Akt phosphorylation without ligand stimulation. Overall, these results suggested that EGFR and Akt activations without ligand stimulation might be partially due to EGFR mutation including amplification. One squamous cell carcinoma had the loss of PTEN protein. Two small-cell carcinoma cell lines with Akt phosphorylation without ligand stimulation, H69 and SBC3, exhibited intermediate sensitivity to gefitinib (Fig. 3). However, they had little EGFR expression and phosphorylation. It is possible that gefitinib may block signal transduction via different receptors including the EGFR family or that SCLC cells may have small amounts of functional EGFR that cannot be detected by Western blotting. H69 had a moderate amount of Her2 expression.

EGF stimulation further increased the phosphorylation of Akt in A549 and PC9 cells, but not in PC14 and ABC-1 cells. A549 and PC9 had EGF responsiveness as well as EGFR and Akt phosphorylation without ligand stimulation. In PC9 cells, the phosphorylation of p44/42 MAP kinase was inhibited at low concentrations of gefitinib. In cell lines with intermediate sensitivity to gefitinib, the phosphorylation of p44/42 MAP kinase was not clearly inhibited, either with or without EGF. These phenomena may be due to differences in activating mechanisms. Lung cancer cells with phosphorylation of p44/42 MAP kinase had no *K-ras *gene mutation other than LCKJ. Han et al reported that only 18.1% of patients with p-Erk positive tumors harbored *K-ras gene *mutation, and identification of other molecular mechanisms leading to p-Erk activation and gefitinib resistance was mandatory [18]. There was no correlation between gefitinib sensitivity, including intermediate-sensitivity, and the status of the *K-ras *gene in our study.

## Conclusion

Our report indicates that sensitivity to gefitinib is related to thephosphorylation of Akt without ligand stimulation. The phosphorylated state of EGFR and Akt might be clinical markers of Akt activation without ligand stimulation and increase specificity of gefitinib sensitivity and, therefore, may prove to be useful prognostic tests of tumor responsiveness, in addition to EGFR gene mutation and amplification. These findings seem to apply especially to adenocarcinomas. Furthermore, EGFR phosphorylation may be an attractive candidate for bioimaging for use in the design of EGFR-targeted therapies.

## Competing interests

There are no financial or other interests with regard to the submitted manuscript that might be construed as a conflict of interest.

## Authors' contributions

RN, AG and SK designed this study analyzed and interpreted the data. RN, YK, SK, YM, KM, KK, MS, TO and AY carried out the cell culture and the sensitivity test. RN, KK, MS and MC carried out analyses of the molecules'expression. and *EGFR *gene status.

## Pre-publication history

The pre-publication history for this paper can be accessed here:



## Figures and Tables

**Table 1:** Phosphorylation and expression of the molecules and their relationship to gefitinib sensitivity

**Expression of the molecules: (+) positive; (-) negative**	*P*
Phosphorylated Akt/Akt (+)	0.0059**
PTEN/β-actin (+)	0.361
phospho-p44/42/p44/42 (+)	0.852
phospho-p38/p38 (+)	>0.99
phospho-EGFR/EGFR (+)	0.0319**
Her2/β-actin (+)	0.0344**
Her3/β-actin(+)	0.5582
phospho-EGFR(+) phospho-Akt(+)	0.0344**
(vs phospho-EGFR(-) or phospho-Akt(-))	

The relationship between gefitinib-sensitivity and the expression of these molecules was investigated by the Chi-square test. **P < 0.05 was considered to be statistically significant.
